# Cognitive Predictors of Word and Pseudoword Reading in Spanish First-Grade Children

**DOI:** 10.3389/fpsyg.2016.00774

**Published:** 2016-05-31

**Authors:** María J. González-Valenzuela, Félix Díaz-Giráldez, María D. López-Montiel

**Affiliations:** ^1^Department of Development and Educational Psychology, University de MálagaMálaga, Spain; ^2^Department of Psychobiology and Methodology of Behavioural Science, University de MálagaMálaga, Spain

**Keywords:** phonological awareness, rapid naming, phonological memory, reading, Spain, primary education

## Abstract

The study examines the individual and combined contribution of several cognitive variables (phonemic awareness, phonological memory, and alphanumeric and non-alphanumeric rapid naming) to word and pseudoword reading ability among first-grade Spanish children. Participants were 116 Spanish-speaking children aged 6 years and without special educational needs, all of whom were attending schools in a medium socioeconomic area. Descriptive/exploratory and bivariate analyses were performed with the data derived from three measures of reading ability (accuracy, speed, and efficiency), and hierarchical multivariate regression models were constructed. In general, the results confirm that, with the exception of non-alphanumeric rapid naming, the cognitive variables studied are predictors of reading performance for words and pseudowords, although their influence differs depending on the reading measures and type of linguistic unit considered. Phonemic awareness, phonological memory, and alphanumeric rapid naming were the best predictors of reading accuracy for words and pseudowords. Variability in the other two measures of reading ability (speed and efficiency) was best explained by alphanumeric rapid naming. These results suggest that reading is a complex skill that depends on different types of cognitive variables according to the age and/or level of the reader, the type of orthography and the type of measure used. Furthermore, they highlight the need to provide instruction in these processes from an early age so as to address or prevent the problems that children may present.

## Introduction

In recent decades a considerable body of research has investigated the variables that determine the acquisition of reading, with most studies finding cognitive correlates of reading ability from an early age (Muter et al., 1998; Manis et al., 2000; Olofsson, 2000; Compton, 2003; Aarnoutse et al., 2005; Georgiou et al., 2006; Silvén et al., 2007; Landerl and Wimmer, 2008; Burke et al., 2009; Moll et al., 2009; Alcock et al., 2010; Babayigit and Stainthorp, 2011; Lei et al., 2011; Li et al., 2012; Nag and Snowling, 2012; Vaessen and Blomert, 2013; Xue et al., 2013). More specifically, phonological awareness, short-term phonological memory, and rapid automatized naming have all been shown to be important, with this being the case for languages of different complexity and linguistic consistency.

Phonological awareness implies the ability to perceive, segment, and manipulate syllables and/or word sounds (Bradley and Bryant, 1983; Wagner and Torgersen, 1987; Vaessen and Blomert, 2013). Rapid automatized naming (RAN) refers to the ability to quickly name visual items, both alphanumeric (letters and numbers) and non-alphanumeric (colors and objects) (Bowers, 1993; Wolf and Denckla, 2003). Rapid automatized naming can involve both phonological and non-phonological skills, since the processes required by a naming task may be visual (detection and discrimination of visual features) and/or phonological (integrating visual information with stored phonological patterns and retrieval of phonological labels) in nature (Wolf and Bowers, 1999). Phonological memory entails the ability to engage in phonological recoding when accessing the lexicon, and it enables the retrieval of stored phonological information (Gathercole et al., 1991; de Jong and van der Leij, 1999; Georgiou et al., 2006). Fewer studies have been conducted into phonological memory than in relation to phonological awareness and RAN, but all three variables have been shown to be associated with reading acquisition and difficulties, since they promote the learning of grapheme-phoneme conversion rules and, therefore, reading accuracy and fluency (Puolakanaho et al., 2008; Kibby, 2009; Kibby et al., 2014).

The double-deficit hypothesis maintains that developmental dyslexias may be due to a deficit in phonological processing, related to difficulties involving phonological memory and/or awareness, or to a deficit in the naming speed for visual stimuli (Wolf and Bowers, 1999; Kibby, 2009; Kibby et al., 2014). In other words, they may be due to a deficit in the manipulation of word sounds or to difficulty accessing and retrieving the names of visual symbols, and hence there are different types of dyslexia (Guzmán et al., 2004). In this context, some studies suggest that naming speed has a direct influence on reading performance, that it is independent of phonological awareness, and that its contribution to word and pseudoword recognition is different to that of phonological awareness (Bowers, 1993; Bowers and Wolf, 1993; Young and Bowers, 1995; Manis et al., 2000; Guzmán et al., 2004; Bowey et al., 2005; Aguilar-Villagrán et al., 2010). However, other authors maintain that the relationship between reading performance and naming speed is an indirect one that is mediated by phonological processing (Näslund and Schneider, 1991; Wagner et al., 1997).

Given the controversy over the relationship between these variables and their repercussions for reading, much of the recent research on predictors of reading performance has sought to determine the combined impact of these cognitive components with respect to different orthographies and different types of reading-related variables, the aim being to establish whether these relationships are stable across different languages and in relation to different aspects of reading. In general, the results indicate that, between the ages of 5 and 8 years, some of these cognitive factors are correlated with reading accuracy and fluency for both words and pseudowords in more transparent or consistent orthographies such as German, Finnish, Norwegian, Dutch, Swedish, Turkish, and Greek (Silvén et al., 2007; Georgiou et al., 2008b; Landerl and Wimmer, 2008; Moll et al., 2009; Babayigit and Stainthorp, 2011; Vaessen and Blomert, 2013), as well as in less transparent or opaque languages such as English, Danish, Chinese or Kannada (Manis et al., 2000; Compton, 2003; Bowey et al., 2005; Moll et al., 2009; Lei et al., 2011; Li et al., 2012; Nag and Snowling, 2012; Xue et al., 2013). Some transcultural studies have also examined concurrent relationships among these variables in subjects from different languages, the aim being to determine which cognitive components have the greatest influence during the first years of school and, therefore, to improve the way in which reading and writing is taught and learnt. The results of these studies differ depending on the orthography or structure of the languages concerned (Defior et al., 2002; Caravolas et al., 2013). Thus, readers of more consistent orthographies gain competence more quickly than do their counterparts in less consistent and/or more opaque languages, with the correlations between reading performance and the different cognitive components being weaker in the case of less consistent languages (Georgiou et al., 2008b; Ziegler et al., 2010). The findings also suggest that the impact of cognitive processing varies depending on which cognitive variables and reading processes are considered, as well as according to the children's age (Seymour et al., 2003; Ziegler et al., 2003, 2010; Georgiou et al., 2008a; Furnes and Samuelsson, 2009; Landerl et al., 2012; Tolchinsky et al., 2012).

Most of the research conducted with Spanish-speaking children has analyzed in a non-concurrent way the relationship between different cognitive correlates and reading performance. These studies have found a strong relationship between phonological awareness (syllabic and phonemic) and the reading of words and pseudowords by children of different ages, this being the case for both dyslexic and normally developing readers (Carrillo, 1994; Jiménez and Ortiz, 2000; Bravo-Valdivieso et al., 2006; Defior, 2008; Rodrigo et al., 2009; Alsina et al., 2011; González-Trujillo et al., 2014; Calet et al., 2015). The observed relationship between phonological awareness and reading performance is directly proportional in younger children, with the effect being greater in relation to phonemic awareness. With respect to naming speed, the results of studies using RAN tasks with Spanish-speaking children are less consistent. Some authors have found that slower naming speeds are associated with more reading difficulties (González-Garrido et al., 2011). Others have found differences in naming speed between dyslexic and normally developing readers, although not between dyslexic subjects and younger children with the same reading age, the conclusion being that dyslexic children with phonological deficits do not present naming-speed problems (Guzmán et al., 2004). As regards the relationship between reading performance and phonological memory, we are aware of no studies with normally developing Spanish-speaking children that have specifically analyzed this aspect. Those studies which have examined phonological memory have done so in conjunction with other variables (Suárez-Coalla et al., 2013) or in samples of dyslexic children (Soriano and Miranda, 2010).

Very few studies have sought to examine the combined effect of different cognitive correlates on the reading performance of Spanish-speaking children. Research in this line has been conducted with preschool children and has found that naming speed and phonological awareness are related to the ability to read words and/or pseudowords (Aguilar-Villagrán et al., 2010; Gómez-Velázquez et al., 2010; Escribano, 2012; González-Seijas et al., 2013; Suárez-Coalla et al., 2013). It should be noted, however, that the results regarding the relative importance of these variables are not always consistent. While some authors report that phonological awareness is a better predictor of word recognition (Escribano, 2012), others maintain that naming speed is more important (Aguilar-Villagrán et al., 2010; Gómez-Velázquez et al., 2010). Furthermore, these relationships vary depending on the reading variables that are considered (accuracy, speed, fluency, or efficiency for words and/or pseudowords), on the measurement index used (number of correct answers during a limited or unlimited period of time, relative or absolute frequencies, etc.), and on whether naming speed is assessed using alphanumeric or non-alphanumeric items (Aguilar-Villagrán et al., 2010; Suárez-Coalla et al., 2013). It also appears that at an early age, phonological memory correlates only with reading accuracy for words and pseudowords when it is considered concurrently with phonological awareness and naming speed (Suárez-Coalla et al., 2013). What is needed, therefore, is an analysis of how those cognitive components that, to date, have been examined in isolation may concurrently impact on the reading ability of Spanish-speaking children of school age. The results obtained could contribute to a better understanding of the processes involved in reading in Spanish and would have important implications for the prevention of reading disabilities.

Consequently, the aim of this study is to examine the individual and combined contribution of several cognitive variables (phonemic awareness, phonological memory, and alphanumeric and non-alphanumeric rapid naming) to word and pseudoword reading ability (measured in terms of accuracy, speed, and efficiency) among first-grade Spanish children.

## Methods

### Participants

Participants were 116 children currently enrolled in the first grade of compulsory education at two schools in a medium socioeconomic area of our city. These schools were selected through stratified random sampling of the official list of schools published by the Regional Board of Education (CEJA, 2013). Regarding the educational level of the children's parents, 17% (*n* = 19) of fathers and 6% (*n* = 7) of mothers had only completed primary school, 65% (*n* = 76) of fathers and 63% (*n* = 73) of mothers had intermediate-level qualifications (secondary/high school or vocational training, and 18% (*n* = 21) of fathers and 31% (*n* = 36) of mothers had received higher education (degree or postgraduate studies).

The sample of children comprised 63 boys (54.3%) and 53 girls (45.7%), aged approximately 6 years (*M* = 79.74 months, *SD* = 3.47). They were all considered to be normal developers, since we excluded any children who had been diagnosed by the school psychology service as having special educational needs. Children originating from other countries and who had yet to develop a good command of Spanish were also excluded.

### Variables and measures

*Word reading accuracy* was assessed using the Word Reading task of the Test of Reading and Writing in Spanish (Defior et al., 2006). This task requires children to read a list of 42 words that have been selected according to their frequency, length, and type of orthographic complexity. Responses are scored from 0 to 2, depending on whether the word is read correctly (2 points), syllable by syllable and/or hesitatingly (1 point), or incorrectly (zero). The total score is the sum of the individual item scores. The task has been shown to be reliable (Cronbach's α = 0.80).

*Word reading speed* was also assessed with the Word Reading task of the Test of Reading and Writing in Spanish (Defior et al., 2006). In this case, the total score for each child was the time that he or she took to read the words included in the list.

*Word reading efficiency* was computed for each child by dividing the word reading accuracy score by the word reading speed score and multiplying the result by 100 (Aguilar-Villagrán et al., 2010).

*Pseudoword reading accuracy* was assessed using the Pseudoword Reading task of the Test of Reading and Writing in Spanish (Defior et al., 2006). This task requires children to read 32 pseudowords that have been selected according to their frequency, length, and type of orthographic complexity. Responses are scored from 0 to 2, depending on whether the word is read correctly (2 points), syllable by syllable and/or hesitatingly (1 point), or incorrectly (zero). The total score is the sum of the individual item scores. The task has been shown to be reliable (Cronbach's α = 0.82).

*Pseudoword reading speed* was also assessed with the Pseudoword Reading task of the Test of Reading and Writing in Spanish (Defior et al., 2006). In this case, the total score for each child was the time that he or she took to read the pseudowords included in the list.

*Pseudoword reading efficiency* was computed for each child by dividing the pseudoword reading accuracy score by the pseudoword reading speed score and multiplying the result by 100 (Aguilar-Villagrán et al., 2010).

*Phonemic awareness* was assessed using a complementary subtest of the Test of Reading and Writing in Spanish (Defior et al., 2006). In this case, children are required to isolate the sounds or letters that make up 14 words, naming the phonemes or letters as they go. The total score corresponds to the number of words that have been correctly segmented. This task has been shown to be reliable (Cronbach's α = 0.91).

*Naming speed* was assessed using a RAN test developed by Wolf and Denckla (2003) and adapted into Spanish by Gómez-Velázquez et al. (2010). This test requires children to name 200 visual stimuli (50 letters, 50 numbers, 50 objects, 50 colors), and we considered two measures of this variable: naming speed for alphanumeric stimuli (letters and numbers) and naming speed for non-alphanumeric stimuli (objects and colors; Compton, 2003; Bowey et al., 2005). The total score for each child was the time that he or she took to name the alphanumeric and non-alphanumeric items, respectively.

*Phonological memory* was assessed using a test of phonological short-term memory developed by Soriano and Miranda (2010), based on Hebrew phonological memory task (Geva et al., 2000). The test requires children to repeat aloud a list of 20 Latin words that are not related to the Spanish lexicon and which do not bear any similarity to Spanish morphology. These words are of different length, and are first pronounced by the examiner. The total score for each child is the number of correct responses. Cronbach's alpha for this test was 0.74.

### Procedure

Based on the official list of schools published by the Regional Board of Education (CEJA, 2013) we randomly selected four schools from among nine in a medium socioeconomic area of our city. Of these four, two schools volunteered to participate in the study.

Following meetings with the corresponding school psychologists we excluded any first-grade children who were classified as having special educational needs or who, due to their originating from another country, had yet to acquire a good command of Spanish. All remaining first-grade students in the two schools were assessed for this study.

Prior to administering the tests, approval was obtained from the Research Ethics Committee of the University of Malaga. The parents or legal guardians of the children also gave their informed consent.

The tests were administered individually by four psychologists across two sessions lasting approximately 20 min each. The word reading task, the pseudoword reading task, and the phonemic awareness test were administered in the first session, while the second involved administration of the RAN test (alphanumeric and non-alphanumeric naming) and the phonological memory test.

### Statistical analysis

Given the objectives of the study we performed descriptive analyses, bivariate analyses in order to explore the relationship between the study variables, and multivariate regression with the aim of explaining the observed variance in scores. For the bivariate analyses we computed Pearson correlation coefficients and the corresponding tests of significance, having first confirmed by means of a graphical analysis that there was a linear relationship between the variables. In order to analyze the combined effect of the cognitive variables on the reading variables, and to determine the specific contribution of each one, we then constructed a series of multivariate regression models for the different performance measures (accuracy, speed, and efficiency) in relation to both word reading and pseudoword reading. For these analyses we included those cognitive variables for which, in the bivariate analysis, the probability associated with Pearson's *r* was less than 0.05.

The most parsimonious and best fitting model for each dependent variable was selected using a backwards procedure guided by the researcher (Kleinbaum et al., 1988; Losilla et al., 2005). Thus, we began with the maximum model that included all the potential explanatory variables and the first-order interaction terms that had been detected in the bivariate analysis, in the order established by the researcher. We then eliminated the non-significant regressor in each successive step, beginning with the interaction effects and continuing with the main effects. Each decision was made by considering the regression coefficients, their statistical significance, the precision (width) of their confidence intervals, and their standard error. The variables retained were then used to construct the most suitable model. The overall significance of the regression models was assessed by means of Fisher's *F*-test, the contrast of the statistical significance of the regression parameters with the Student's *t*-test (two-tailed). In order to evaluate globally the proportion of the variance in each dependent variable that could be attributed to the regression model we computed the coefficient of determination (*R*^2^) and the adjusted coefficient of determination (${\bar{R}}^{2}$). To determine the contribution of each explanatory variable to the total variance in the dependent variable we calculated the semi-partial correlation coefficient (${sr}_{i}^{2}$). The practical significance of the estimated models was evaluated by calculating the *f*^2^ statistic as a measure of effect size in the correlation family. The fit of the data to the assumptions of the linear regression model was tested a posteriori by means of a diagnostic plot of residuals. Multicollinearity was examined by computing the variance inflation factor (VIF), the values of which were all smaller than 10, indicating that multicollinearity was not very serious.

All data processing and statistical analysis was performed using SPSS v22.

## Results

Table 1 summarizes the descriptive analysis and the correlations between the study variables, along with their statistical significance.

**Table 1:** **Descriptive statistics and correlations**.

**Variables**	**Mean**	***SD***	**Range**	**1**	**2**	**3**	**4**	**5**	**6**	**7**	**8**	**9**	**10**
1. WRA	59.03	11.82	20–81	–									
2. WRS	92.14	29.38	44–194	−0.65^**^	–								
3. WRE	73.09	32.40	13.92–172.73	0.82^**^	−0.88^**^	–							
4. PRA	53.16	11.39	16–77	0.82^**^	−0.63^**^	0.70^**^	–						
5. PRS	99.17	26.31	48–186	−0.52^**^	0.88^**^	0.75^**^	−0.52^**^	–					
6. PRE	58.75	23.14	9.68–143.75	0.71^**^	−0.82^**^	0.88^**^	0.79^**^	0.85^**^	–				
7. PA	8.34	2.40	1–14	0.42^**^	−0.22^**^	0.33^**^	0.30^**^	−0.12	0.22^*^	–			
8. PM	16.43	2.33	10–20	0.33^**^	−0.14	0.20^*^	0.25^**^	−0.10	0.14	0.28^**^	–		
9. ARN	74.17	14.60	45–126	−0.24^**^	0.46^**^	0.41^**^	−0.26^**^	0.46^**^	−0.44^**^	−0.09	0.08	–	
10. NARN	179.37	41.43	91–333	−0.25^**^	0.29^**^	0.26^**^	−0.29^**^	0.23^*^	−0.25^**^	−0.17	−0.18	0.31^*^	–

** Pearson r correlation coefficient significant at p < 0.01;

* *Pearson r correlation coefficient significant at p < 0.05*.

*SD, standard deviation; WRA, word reading accuracy (n° of correct responses); WRS, word reading speed (seconds); WRE, word reading efficiency (WRA/WRS × 100); PRA, pseudoword reading accuracy (n° of correct responses); PRS, pseudoword reading speed (seconds); PRE, pseudoword reading efficiency (PRA/PRS × 100); PA, phonemic awareness (n° of correct responses); PM, phonological memory (n° of correct responses); ARN, alphanumeric rapid naming (seconds); NARN, non-alphanumeric rapid naming (seconds)*.

Analysis of the associations between the cognitive variables and the reading variables revealed a significant relationship between almost all of them (see Table 1). For both words and pseudowords, reading accuracy was more strongly correlated with phonemic awareness, whereas reading speed and efficiency showed stronger correlations with alphanumeric rapid naming. However, no significant relationship was observed between the following pairs of variables: word reading speed and phonological memory; pseudoword reading speed and phonemic awareness; pseudoword reading speed and phonological memory; or pseudoword reading efficiency and phonological memory. As regards the bivariate relationships between the phonological variables considered in this study, we observed a statistically significant association between phonemic awareness and phonological memory (see Table 1).

In order to examine the contribution of the cognitive variables (phonemic awareness, phonological memory, and alphanumeric and non-alphanumeric rapid naming) to word and pseudoword reading (measured in terms of accuracy, speed, and efficiency) we then performed a series of hierarchical regressions. In the first model estimated for each dependent variable we included all those theoretically plausible variables for which, in the bivariate analysis, the probability associated with Pearson's *r* was less than 0.05. Any significant interactions between the independent variables were also included. Starting from this initial model, the independent variables were then eliminated one at a time in an order determined by the researcher. In general, all the models for each dependent variable were statistically significant, and no final model included the interaction effects introduced in the initial model. In the following sections we present the results for word and pseudoword reading accuracy, speed, and efficiency according to the final models selected and having checked through an analysis of residuals that the data fitted the assumptions of the linear regression model (see Tables 2, 3).

**Table 2:** **Multivariate regression analysis for word reading**.

	***Variables***	***B***	***SE***	**β**	***t***	***p***	***sr***	***VIF***
**WRA**								
Model 1	Intercept	19.19	19.79		0.98	0.334		
	PA	5.03	2.53	1.15	1.99	0.049	0.16	
	PM	2.76	1.16	0.56	2.38	0.019	0.19	
	ARN	−0.16	0.07	−0.20	−2.38	0.019	−0.19	
	NARN	−0.03	0.03	−0.09	−1.07	0.289	−0.09	
	PA × PM	−0.220	0.15	0.97	−1.45	0.149	−0.12	
^a^*F*_(5, 110)_ = 9.27, *p* = 0.001; *R*^2^ = 0.29; adjusted *R*^2^ = 0.26; *R* = 0.54								
Model 2	Intercept	44.69	9.17		4.88	0.000		
	PA	1.39	0.37	0.32	3.76	0.000	0.30	
	PM	1.19	0.42	0.24	2.82	0.006	0.23	
	ARN	−0.17	0.07	−0.21	−2.42	0.017	−0.19	
	NARN	−0.03	0.03	−0.09	−0.99	0.321	−0.08	
^a^*F*_(4, 111)_ = 10.95, *p* = 0.000; *R*^2^ = 0.28; adjusted *R*^2^ = 0.26; *R* = 0.53								
Model 3	Intercept	40.34	8.06		5.01	0.000		
	PA	1.43	0.37	0.33	3.87	0.000	0.31	1.09
	PM	1.27	0.41	0.26	3.06	0.003	0.25	1.09
	ARN	−0.19	0.07	−0.23	−2.88	0.005	−0.23	1.02
^a^*F*_(3, 112)_ = 14.27, *p* = 0.000, ^b^*f*^2^ = .42; *R*^2^ = 0.28; adjusted *R*^2^ = 0.26; *R* = 0.53								
**WRS**								
Model 1	Intercept	28.04	17.09		1.64	0.104		
	ARN	0.81	0.17	0.40	4.74	0.000	0.38	
	PA	−1.76	0.89	−0.16	−1.97	0.052	−0.16	
	NARN	0.10	0.06	0.15	1.70	0.092	0.14	
^a^*F*_(3, 112)_ = 13.46, *p* = 0.001; *R*^2^ = 0.27; adjusted *R*^2^ = 0.25; *R* = 0.52								
Model 2	Intercept	42.15	15.06		2.79	0.006		
	ARN	0.89	0.17	0.46	5.44	0.000	0.44	1.01
	PA	−1.99	0.89	−0.18	−2.23	0.028	−0.18	1.01
^a^*F*_(2, 113)_ = 18.44, *p* = 0.000, ^b^*f*^2^ =.32; *R*^2^ = 0.25; adjusted *R*^2^ = 0.23; *R* = 0.49								
**WRE**								
Model 1	Intercept	80.97	54.73		1.48	0.142		
	ARN	−0.83	0.19	−0.38	−4.36	0.000	−0.35	
	PA	3.96	6.99	0.33	0.57	0.572	0.05	
	PM	2.45	3.20	0.18	0.77	0.446	0.06	
	NARN	−0.06	0.07	−0.08	−0.88	0.380	−0.07	
	PA × PM	−0.06	0.42	−0.10	−0.15	0.882	−0.01	
^a^*F*_(5, 110)_ = 8.68, *p* = 0.001; *R*^2^ = 0.28; adjusted *R*^2^ = 0.25; *R* = 0.53								
Model 2	Intercept	88.19	25.12		3.51	0.001		
	ARN	−0.83	0.19	−0.38	−4.39	0.000	−0.35	
	PA	2.93	1.02	0.24	2.88	0.005	0.23	
	PM	2.01	1.16	0.15	1.73	0.085	0.14	
	NARN	−0.06	0.07	−0.08	−0.88	0.381	−0.07	
^a^*F*_(4, 111)_ = 10.94, *p* = 0.000; *R*^2^ = 0.28; adjusted *R*^2^ = 0.26; *R* = 0.53								
Model 3	Intercept	77.66	22.06		3.52	0.001		
	ARN	−0.89	0.18	−0.39	−4.92	0.000	−0.39	
	PA	3.01	1.01	0.25	2.98	0.004	0.24	
	PM	2.19	1.13	0.16	1.93	0.056	0.16	
^a^*F*_(3, 112)_ = 14.36, *p* = 0.000; *R*^2^ = 0.28; adjusted *R*^2^ = 0.26; *R* = 0.53								
Model 4	Intercept	106.32	16.53		6.43	0.000		
	ARN	−0.85	0.18	−0.38	−4.69	0.000	−0.38	1.01
	PA	3.57	0.98	0.30	3.64	0.000	0.30	1.01
^a^*F*_(2, 113)_ = 19.21, *p* = 0.000, *f*^2^ = 0.34; *R*^2^ = 0.25; adjusted *R*^2^ = 0.24; *R* = 0.50								

*WRA, word reading accuracy; WRS, word reading speed; WRE, word reading efficiency; PA, phonemic awareness; PM, phonological memory; ARN, alphanumeric rapid naming; NARN, non-alphanumeric rapid naming; SE, standard error; sr, semi-partial correlation; VIF, variance inflation factor*.

a *Goodness-of-fit tests for multivariate regression models: Global test F, coefficient of determination; R^2^, adjusted coefficient of determination; adjusted R^2^*.

b *f^2^ = Index of effect size in the correlation family (reference values: small = 0.02; medium = 0.15; large = 0.35)*.

**Table 3:** **Multivariate regression analysis for pseudoword reading**.

	***Variables***	***B***	***SE***	**β**	***t***	***p***	***sr***	***VIF***
**PARA**								
Model 1	Intercept	34083	20.13		1.73	0.086		
	ARN	−0.16	0.07	−0.21	−2.28	0.025	−0.19	
	PA	3.30	2.57	0.78	1.29	0.202	0.11	
	PM	1.90	1.19	0.40	1.61	0.109	−0.14	
	NARN	−0.05	0.03	−0.17	−1.82	0.072	−0.15	
	PA x PM	−0.15	0.16	−0.68	−0.96	0.341	−0.08	
^a^*F*_(5, 110)_ = 6.02, *p* = 0.001; *R*^2^ = 0.22; adjusted *R*^2^ = 0.18; *R* = 0.46								
Model 2	Intercept	51.91	9.28		5.59	0.000		
	ARN	−0.16	0.07	−0.21	−2.31	0.023	−0.19	
	PA	0.87	0.38	0.21	2.32	0.022	0.19	
	PM	0.85	0.43	0.18	1.99	0.048	0.17	
	NARN	−0.05	0.02	−0.16	−1.78	0.078	−0.15	
^a^*F*_(4, 111)_ = 7.30, *p* = 0.000; *R*^2^ = 0.21; adjusted *R*^2^ = 0.18; *R* = 0.46								
	Intercept	44.04	8.23		5.34	0.000		
Model 3	ARN	−0.20	0.07	−0.26	−2.99	0.003	−0.26	1.02
	PA	0.93	0.38	0.22	2.47	0.015	0.21	1.01
	PM	0.99	0.42	0.21	2.34	0.021	0.20	1.09
^a^*F*_(3, 112)_ = 8.51, *p* = 0.000, ^b^*f*^2^ = 0.23; *R*^2^ = 0.19; adjusted *R*^2^ = 0.16; *R* = 0.43								
**PRS**								
Model 1	Intercept	31.24	13.04		2.39	0.018		
	ARN	0.77	0.16	0.43	4.87	0.000	0.41	
	NARN	0.06	0.06	0.09	1.11	0.271	0.09	
^a^*F*_(2, 113)_ = 15.53, *p* = 0.000; *R*^2^ = 0.22; adjusted *R*^2^ = 0.20; *R* = 0.46								
	Intercept	38.20	11.37		3.37	0.001		
Model 2	ARN	0.82	0.15	0.46	5.46	0.000	0.46	
^a^*F*_(1, 114)_ = 29.77, *p* = 0.000; ^b^*f*^2^ = 0.26; *R*^2^ = 0.21; *R* = 0.46								
**PRE**								
Model 1	Intercept	103.32	13.73		7.53	0.000		
	ARN	−0.63	0.14	−0.39	−4.57	0.000	−0.38	
	PA	1.43	0.72	0.17	1.98	0.050	0.16	
	NARN	−0.06	0.05	−0.09	−1.12	0.267	−0.09	
^a^*F*_(3, 112)_ = 11.47, *p* = 0.000; *R*^2^ = 0.24; adjusted *R*^2^ = 0.22; *R* = 0.49								
Model 2	Intercept	95.88	12.02		7.98	0.000		
	ARN	−0.67	0.13	−0.43	−5.12	0.000	−0.42	1.01
	PA	1.55	0.71	0.18	2.17	0.032	0.18	1.01
^a^*F*_(2, 111)_ = 16.55, *p* = 0.000,^b^*f*^2^ = 0.29; *R*^2^ = 0.23; adjusted *R*^2^ = 0.21; *R* = 0.48								

*PRA, pseudoword reading accuracy; PRS, pseudoword reading speed; PRE, pseudoword reading efficiency; PA, phonemic awareness; PM, phonological memory; ARN, alphanumeric rapid naming; NARN, non-alphanumeric rapid naming; SE, standard error; sr, semi-partial correlation; VIF, variance inflation factor*.

a *Goodness-of-fit tests for multivariate regression models: Global test F, coefficient of determination; R^2^, adjusted coefficient of determination; adjusted R^2^*.

b *f^2^ = Index of effect size in the correlation family (reference values: small = 0.02; medium = 0.15; large = 0.35)*.

### Word reading

With word reading accuracy as the dependent variable the regression procedure yielded a final model with a good overall fit [*F*_(3, 112)_ = 14.27, *p* < 0.001, *f*^2^ = 0.42] and which included the following explanatory variables: phonemic awareness [*t*_(115)_ = 3.87, *p* < 0.001], phonological memory [*t*_(115)_ = 3.06, *p* < 0.01], and alphanumeric rapid naming [*t*_(115)_ = −2.88, *p* < 0.01]. The coefficient of determination (adjusted ${\bar{R}}^{2}=0.26$) indicated that 26% of the variability in the response variable was explained by the regression model. Based on the corresponding semi-partial correlation coefficients (${sr}_{i}^{2}$), phonemic awareness contributed 9.6%, phonological memory 6.25%, and alphanumeric rapid naming 2%.

With word reading speed as the dependent variable the final fitted model [*F*_(2, 113)_ = 18.44, *p* < 0.001, *f*^2^ = 0.32] included the variables alphanumeric rapid naming [*t*_(115)_ = 5.44, *p* < 0.001] and phonemic awareness [*t*_(115)_ = −2.23, *p* < 0.05], and explained 23% of the variance in the response variable (adjusted ${\bar{\text{R}}}^{2}=0.23$). The corresponding semi-partial correlation coefficients (${sr}_{i}^{2}$) indicated that alphanumeric rapid naming contributed 19.36% and phonemic awareness 3.24%.

Finally, with word reading efficiency as the dependent variable the final regression model selected [*F*_(2, 113)_ = 19.21, *p* < 0.00, *f*^2^ = 0.34] included the main effects of two variables, alphanumeric rapid naming [*t*_(115)_ = −4.69, *p* < 0.001] and phonemic awareness [*t*_(115)_ = 3.64, *p* < 0.001], explaining 24% of the variance in the dependent variable (adjusted ${\bar{R}}^{2}$ = 0.24). Based on the corresponding semi-partial correlation coefficients (${sr}_{i}^{2}$), alphanumeric rapid naming contributed 14.44% and phonemic awareness 9%.

### Pseudoword reading

With pseudoword reading accuracy as the dependent variable the final estimated model [*F*_(3, 112)_ = 8.51, *p* < 0.001, *f*^2^ = 0.23] included the variables alphanumeric rapid naming [*t*_(115)_ = −2.99, *p* < 0.01], phonemic awareness [*t*_(115)_ = 2.47, *p* < 0.05], and phonological memory [*t*_(115)_ = 2.34, *p* < 0.05], and it explained 16% of the variance in the response variable (adjusted ${\bar{R}}^{2}$ = 0.16). The corresponding semi-partial correlation coefficients (${sr}_{i}^{2}$) indicated that alphanumeric rapid naming contributed 6.76%, phonemic awareness 4.41%, and phonological memory 4%.

With pseudoword reading speed as the dependent variable the final model [*F*_(1, 114)_ = 29.77, *p* < 0.001, *f*^2^ = 0.26] included just one independent variable, alphanumeric rapid naming [*t*_(115)_ = 5.46, *p* < 0.001], which explained 21% of the variance in the outcome variable (*R*^2^ = 0.21).

Finally, with pseudoword reading efficiency as the response variable the final fitted model [*F*_(2, 111)_ = 16.55, *p* < 0.001, *f*^2^ = 0.29] included two explanatory variables, alphanumeric rapid naming [*t*_(115)_ = −5.12, *p* < 0.001] and phonemic awareness [*t*_(115)_ = 2.17, *p* < 0.05], explaining 21% of the variance in the dependent variable (adjusted ${\bar{R}}^{2}=0.21$). The corresponding semi-partial correlation coefficients (${sr}_{i}^{2}$) indicated that alphanumeric rapid naming contributed 17.64% and phonemic awareness 3.24%.

## Discussion

The aim of this study was to determine the extent to which the word and pseudoword reading ability (measured in terms of accuracy, speed, and efficiency) of Spanish first-grade children could be explained by a series of potentially relevant cognitive variables, namely phonemic awareness, phonological memory, and alphanumeric and non-alphanumeric rapid naming. We examined both the individual contribution of each of these variables, as well as their combined influence.

The analysis revealed that almost all the independent variables considered had an effect on reading performance, and that together they explained a substantial and significant proportion of the variance observed among children. Importantly, however, the relative contribution of each variable differed depending on the task and the measure of reading ability that was used.

For Spanish readers of the ages considered here, non-alphanumeric rapid naming (of objects and colors) explained none of the reading variables examined. This finding is consistent both with previous studies of 6-year-old children in different languages, which likewise found no explanatory role for object naming in relation to word and pseudoword reading ability (Olofsson, 2000; Aguilar-Villagrán et al., 2010), as well as with research showing that alphanumeric rapid naming is a stronger predictor of reading performance than is non-alphanumeric naming (Cronin and Carver, 1998; Compton, 2003; Cardoso-Martins and Pennington, 2004; Bowey et al., 2005; Aguilar-Villagrán et al., 2010). However, our results do not coincide with those of some other studies in this field which found that rapid naming of objects and/or colors was a good predictor (1) of reading speed in 5-year-old Spanish readers (Suárez-Coalla et al., 2013), (2) of word reading accuracy and pseudoword reading speed and efficiency in 5-year-old Spanish readers (Aguilar-Villagrán et al., 2010), (3) of word reading fluency at age 9 in German readers when measured at age 6 (Landerl and Wimmer, 2008), and (4) of word reading fluency and recognition of Chinese characters at age 8 when measured at the pre-school stage (Lei et al., 2011). These differences with respect to our findings could be due to the fact that rapid naming of objects and colors was considered in terms of efficiency rather than speed in some of these previous studies (Aguilar-Villagrán et al., 2010), while in others these variables were studied individually rather than together as we did here (Landerl and Wimmer, 2008; Aguilar-Villagrán et al., 2010; Lei et al., 2011; Suárez-Coalla et al., 2013). It should be noted that some authors maintain that, given the relationship of each component to reading performance, it is advisable to separate alphanumeric components from non-alphanumeric ones, since their relative contribution to the process may be similar and/or different depending on which aspect of the reading process is being examined (Cronin and Carver, 1998; Compton, 2003; Cardoso-Martins and Pennington, 2004; Bowey et al., 2005). The differences between our results and some of the literature could also be because the importance of these non-alphanumeric components varies according to age and the characteristics of the language being studied, as other authors have previously suggested (Cardoso-Martins and Pennington, 2004; Lervag and Hulme, 2009). In this regard it should be noted that in some of these previous studies the children were pre-readers (Aguilar-Villagrán et al., 2010; Suárez-Coalla et al., 2013), while in others they were readers of languages which are less consistent than is Spanish (Landerl and Wimmer, 2008; Lei et al., 2011).

As regards alphanumeric rapid naming (of letters and numbers) our analysis showed that this was related to all the reading variables considered. More specifically, alphanumeric rapid naming explained the highest proportion of the variance not only in word reading speed and efficiency, but also in pseudoword reading accuracy, speed, and efficiency. These results are in line with most of the studies that have used letters and/or numbers as a measure of RAN, with a strong relationship being observed between this measure and both word and pseudoword reading performance in different languages (Bowers and Newby-Clark, 2002; Neuhaus and Swank, 2002; Compton, 2003; Aarnoutse et al., 2005; Cirino et al., 2005; Georgiou et al., 2008a; Moll et al., 2009; Papadopoulos et al., 2009; Vaessen et al., 2009; Aguilar-Villagrán et al., 2010; Babayigit and Stainthorp, 2011; Lei et al., 2011; Nag and Snowling, 2012; Vaessen and Blomert, 2013; Xue et al., 2013). Our results are also consistent with the double-deficit hypothesis of dyslexia, whereby children with a slower naming speed would have greater difficulties identifying and retrieving the phonological representation, leading them to be slower and less accurate readers (Wolf and Bowers, 1999; Suárez-Coalla et al., 2013). Other studies, conducted in different languages and with children of different ages, have suggested that alphanumeric rapid naming is more strongly related to reading speed and fluency (correct answers per minute) or to efficiency (correct answers in relation to the time taken) for both words and pseudowords (Aarnoutse et al., 2005; Moll et al., 2009; Aguilar-Villagrán et al., 2010; Gómez-Velázquez et al., 2010; Babayigit and Stainthorp, 2011; Lei et al., 2011; Xue et al., 2013). Conversely, other studies report a stronger relationship with respect to word reading accuracy (Manis et al., 2000; Compton, 2003; Li et al., 2012; Nag and Snowling, 2012). Our results indicate that the contribution of alphanumeric rapid naming is slightly greater in relation to reading speed and efficiency. This could be because these reading variables are based on time units, as is alphanumeric rapid naming, such that children with faster naming speeds for letters and numbers are also those who show greater fluency when reading words and pseudowords (Wolf and Bowers, 1999). It should also be noted that in our study the relationship with alphanumeric rapid naming was similar for both word and pseudoword reading, in contrast to some studies which report a stronger relationship with word reading (Manis et al., 2000; Aguilar-Villagrán et al., 2010). This discrepancy could be due to differences in the way in which alphanumeric rapid naming was measured, since Aguilar-Villagrán et al. (2010) based their measure on reading efficiency, or to the characteristics of the language being studied, since the study by Manis et al. (2000) involved readers of English, a less consistent language than is Spanish.

With respect to phonemic awareness our analysis showed that this was also related, in general, to all the reading variables. Furthermore, it was the cognitive variable that explained the greatest proportion of the variance in word reading accuracy. This finding is consistent with the results of other studies in Spanish that have likewise highlighted the importance of phonemic awareness for the word and pseudoword reading ability of young children (Jiménez and Ortiz, 2000; Defior et al., 2006; Aguilar-Villagrán et al., 2010; Escribano, 2012; Suárez-Coalla et al., 2013). Research conducted in languages both more and less consistent than Spanish also shows that phonemic awareness plays an important role at different ages and in relationship to different reading variables (such as accuracy, fluency, and efficiency), since it is a process that favors children's acquisition of grapheme-phoneme conversion rules (Manis et al., 2000; Olofsson, 2000; Silvén et al., 2007; Georgiou et al., 2008a; Furnes and Samuelsson, 2009; Alcock et al., 2010; Babayigit and Stainthorp, 2011; Landerl et al., 2012; Li et al., 2012; Nag and Snowling, 2012; Vaessen and Blomert, 2013; Xue et al., 2013). In the present study we found that phonemic awareness was most strongly related to reading accuracy and reading efficiency, and also that its effect was greater for words than for pseudowords, findings which are consistent with some previous studies of 5-year-old Spanish readers (Aguilar-Villagrán et al., 2010; Suárez-Coalla et al., 2013). On the one hand, these results demonstrate the relationship between phonemic awareness and the development of grapheme-phoneme conversion rules, and highlight how such awareness helps children acquire good accuracy when reading. On the other hand, however, it is paradoxical that phonemic awareness correlates more strongly with reading performance for words as opposed to pseudowords in a transparent language like Spanish. A possible explanation for this finding is that children of the age considered here have yet to develop fully the ability to engage in phonological processing, the skill most commonly employed in consistent orthographies (Landerl et al., 2012). We also found that the relationship between phonemic awareness and reading variables was weaker than that observed for rapid automatized naming, a result that contrasts with the findings of Escribano (2012) and Vaessen and Blomert (2013) for reading fluency at age six, and those of Olofsson (2000) and Suárez-Coalla et al. (2013) for reading accuracy at age five. These discrepancies could be due to the way in which rapid naming and/or the components considered were measured. Our results are, however, in line with most of the studies conducted at different ages, in different languages, and with different reading variables, since rapid automatized naming is generally reported to be the best predictor of word and pseudoword reading accuracy, fluency, or efficiency, not only in Spanish but also in languages both more and less consistent in terms of orthography (Compton, 2003; Aarnoutse et al., 2005; Georgiou et al., 2008a; Moll et al., 2009; Aguilar-Villagrán et al., 2010; Gómez-Velázquez et al., 2010; Lei et al., 2011; Li et al., 2012; Nag and Snowling, 2012; Vaessen and Blomert, 2013). Our findings also reflect those studies on the rapid acquisition of phonological skills and reading which have concluded that phonemic awareness becomes less relevant as children become better readers (Seymour et al., 2003; Cuetos and Suárez-Coalla, 2009; Suárez-Coalla et al., 2013; Vaessen and Blomert, 2013).

As regards phonological memory, our analysis suggests that this only has an influence on reading accuracy for words and pseudowords. These results are similar to those reported by Suárez-Coalla et al. (2013) for word and pseudoword reading accuracy among 5-year-old Spanish readers, as well as those obtained for word reading accuracy by Georgiou et al. (2008a) in their study of Greek children aged six and seven, and by Kibby et al. (2014) in a sample of North American children with dyslexia aged 8–12 years. Landerl and Wimmer (2008), in a study of 6-year-old English readers, and Alcock et al. (2010), with 6- and 7-year-old Swahili readers, likewise failed to find a relationship between phonological memory and, respectively, word reading fluency and letter reading accuracy. These results coincide with those of other studies showing that phonological memory is more closely related to developmental dyslexias (Caravolas et al., 2005; Georgiou et al., 2008a), and highlight the discrepancies between the findings of different studies. Some authors claim that phonological memory does contribute to reading ability (Swanson and Howell, 2001; Puolakanaho et al., 2008; Kibby, 2009; Kibby et al., 2014), whereas others argue that it is only weakly related when considered in conjunction with phonological awareness and naming speed (Dufva et al., 2001; Parrilla et al., 2004).

As very few studies with Spanish readers have examined the combined contribution of phonological awareness, naming speed, and phonological memory to the word and pseudoword reading accuracy, speed, and efficiency of children in the first years of schooling, it is difficult to make direct comparisons between our data and previous research. Some of our findings are similar to those reported in the literature for 5- and 6-year-old readers in Spanish and/or other languages, while others are discrepant. It should be noted, however, that some of these studies did not consider phonological memory or pseudoword reading, and different measures were used to evaluate the study variables included here. Furthermore, not all previous studies have examined the same cognitive or reading variables as we did.

It is important to note that a variety of tasks have been used by researchers to assess reading performance, and also that different studies have used different empirical measures. The most widely used task involves reading a list of words, while lists of pseudowords or whole texts are less frequently employed. The most common measures of reading ability are based on accuracy, fluency, and efficiency. Reading accuracy is usually measured in terms of relative or absolute frequencies, or the number of correct responses or errors as a proportion of the total. Fluency tends to be based on the number of correct responses or errors over a fixed time period (usually 60 or 45 s). As a measure of reading efficiency, researchers generally use a compound measure that considers both time and the number of correct responses. The tasks and measures used to evaluate phonemic awareness and naming speed also vary across studies. In the case of phonemic awareness, most studies use tasks based on the omission or discrimination of the initial phoneme, although others employ tasks involving the addition or omission of syllables and phonemes, the deletion and substitution of phonemes, rhymes, or the isolation, synthesis, and segmentation of phonemes. As regards naming speed, this is sometimes evaluated using just one or more of the four traditional components (i.e., letters, numbers, objects, and colors), although it should also be noted that the alphanumeric and non-alphanumeric items are not always examined separately. These differences make it difficult to draw firm conclusions and hamper the comparison of results obtained by different studies, not only those conducted in different languages but also those in the same language. This highlights the need for systematic reviews and meta-analyses regarding the methodological characteristics of studies that have analyzed the contribution of cognitive factors to reading performance.

In general, the results of our study indicate that the cognitive variables considered (phonemic awareness, alphanumeric naming speed, and phonological memory) are predictors of word and pseudoword reading ability among Spanish first-grade children, although their influence differs according to the reading measure and type of linguistic unit considered. Phonemic awareness, alphanumeric naming speed, and phonological memory all contributed to the variability in word and pseudoword reading accuracy, with the effect being greater for words. The variability in the other reading measures (speed and efficiency) was best explained by alphanumeric naming speed, with the effect again being greater for words. This suggests that children who are quicker at naming letters and numbers will be faster on reading tasks, and if, in addition, they have better phonemic awareness and phonological memory skills then they will also achieve greater accuracy on these tasks during the early stages of their learning. Discrepancies between some of our findings and the literature are attributed, first, to the fact that very few studies have examined all these variables simultaneously among first-grade Spanish children, and second, to the lack of homogeneity among the tasks and empirical measures used to assess reading performance. These results suggest that reading is a complex skill that depends on the age and/or level of the reader, the type of orthography, and the type of task used to measure reading. They also highlight the need to provide instruction in these processes from an early age so as to address or prevent the problems that children may present. However, they should be considered with caution because the sample was homogeneous in SES composition (middle class) and geographical background (urban). Thus, the relatively homogenous simple might limit the generalizability of the results. In future studies a larger sample with different SES levels and geographical backgrounds may be needed to further explore the concurrent predictors of word and pseudoword reading.

Finally, future studies on this question could also take their lead from transcultural research involving children with dyslexia (Landerl et al., 2012) or their normally developing peers in different languages (Georgiou et al., 2008b; Furnes and Samuelsson, 2009; Tolchinsky et al., 2012) and examine whether, in the early stages of literacy, the effect of these cognitive variables on the different aspects of reading performance is the same in Spanish as it is in other less consistent languages.

## Author contributions

MG have contributed to the conception and design of work, to interpretation of data, drafting the article and revising it critically for important intellectual content, a final approval of the version to be published, and to agreement to be accountable for all aspects of the work in ensuring that questions related to the accuracy or integrity of any part of the work are appropriately investigated and resolved. ML have contributed to analysis and interpretation of data, drafting the article or revising it critically for important intellectual content, a final approval of the version to be published, and to agreement to be accountable for all aspects of the work in ensuring that questions related to the accuracy or integrity of any part of the work are appropriately investigated and resolved. FD have contributed to acquisition of data, to interpretation of data, drafting the article or revising it critically for important intellectual content, a final approval of the version to be published to be published, and to agreement to be accountable for all aspects of the work in ensuring that questions related to the accuracy or integrity of any part of the work are appropriately investigated and resolved.

### Conflict of interest statement

The authors declare that the research was conducted in the absence of any commercial or financial relationships that could be construed as a potential conflict of interest.
